# A cross-sectional study of the associations between the traditional Japanese diet and nutrient intakes: the NILS-LSA project

**DOI:** 10.1186/s12937-019-0468-9

**Published:** 2019-07-30

**Authors:** Shu Zhang, Rei Otsuka, Yasutake Tomata, Hiroshi Shimokata, Chikako Tange, Makiko Tomida, Yukiko Nishita, Sanae Matsuyama, Ichiro Tsuji

**Affiliations:** 1 Division of Epidemiology, Department of Health Informatics and Public Health, Tohoku University School of Public Health, Graduate School of Medicine, 2-1 Seiryo-machi, Aoba-ku, Sendai, Miyagi 980-8575 Japan; 2 Section of NILS-LSA, National Center for Geriatrics and Gerontology, 7-430 Morioka-cho, Obu City, Aichi 474-8511 Japan; 3 Institute of Health and Nutrition, Nagoya University of Arts and Sciences, Takeno-yama 57 Iwasaki-cho, Nisshin City, Aichi 470-0196 Japan; 4 Department of Epidemiology of Aging, National Center for Geriatrics and Gerontology, Obu City, Aichi 474-8511 Japan

**Keywords:** Japanese diet, Nutrient intake, Nutrient density, Dietary record, Cross-sectional study

## Abstract

**Background:**

Although our previous study using a food frequency questionnaire simulated nutritional characteristics of the traditional Japanese diet, this issue has not been sufficiently evaluated. This study was conducted to examine the relationship between the traditional Japanese diet and nutrient density (ND).

**Methods:**

A cross-sectional study employing the dietary record method was conducted among 2221 community-dwelling Japanese adults (40–88 years) living in Aichi Prefecture, Japan, in 2006–2008. Based on previous studies, a 9-component Japanese Diet Index (JDI) and a 12-component modified JDI (mJDI_12_) were defined. To develop a new weighted index, a multiple linear regression model was used to select food components which were significantly associated with an ND score (integrated by 11 nutrient components) from the mJDI_12_ and weight them. Correlation analyses were performed between JDI, mJDI_12_, the new weighted JDI score and the ND score and its 11 nutrient components. The findings were validated with data from 2008 to 2010 by assessing the associations between the JDIs scores and the ND score.

**Results:**

Scores of the JDI and mJDI_12_ were positively correlated with the ND score (corresponding Spearman’s ρ [95% confidence interval; CI], 0.34 [0.31, 0.38] and 0.44 [0.41, 0.48], respectively; *P* < 0.05 for both). Among the mJDI_12_, 9 food components (rice, fish and shellfish, green and yellow vegetables, seaweed, green tea, beef and pork, soybeans and soybean foods, fruit, and mushrooms) significantly associated with the ND score. All of these 9 components were weighted and a new weighted JDI (wJDI_9_) was developed. The wJDI_9_ score was also positively correlated with the ND score (Spearman’s ρ [95% CI] = 0.61 [0.58, 0.64]; *P* < 0.05). However, scores for all 3 indices were positively correlated with sodium intake. The wJDI_9_ score obtained using dietary record data from 2008 to 2010 was also positively correlated with the ND score (Spearman’s ρ [95% CI] = 0.61 [0.58, 0.64]; *P* < 0.05).

**Conclusions-:**

Adhering to a traditional Japanese diet as defined by the JDI was associated with good ND. Furthermore, the modified indices (mJDI_12_ and wJDI_9_) had a higher performance for ND. However, all of the indices were correlated with high sodium intake.

**Electronic supplementary material:**

The online version of this article (10.1186/s12937-019-0468-9) contains supplementary material, which is available to authorized users.

## Background

Previous studies have reported that a traditional Japanese diet is associated with a lower risk of mortality [1–3] and adverse health outcomes (e.g. disability, dementia, depression) [4–7]. Although such findings have suggested that the traditional Japanese diet well balanced in terms of nutrient intake, the nutritional characteristics of the traditional Japanese diet have remained unclear.

According to the Japanese Diet Index (JDI) [8], 7 adhering components (“rice”, “miso soup”, “fish and shellfish”, “green and yellow vegetables”, “seaweed”, “pickles”, “green tea”) and 2 non-adhering components (“beef and pork”, “coffee”) are considered part of the traditional Japanese diet. In our previous study [4], the JDI was derived by factor analysis (principal component analysis; PCA) and confirmatory factor analysis by using the daily consumption (weight in grams) of 39 food items from the FFQ. Recent studies [9–11] also showed consistency in regard to the food components with the JDI with other typical Japanese dietary patterns derived by PCA or dietary records (DRs).

Our previous simulation study suggested that the JDI is related to better overall nutrient intake [8]. In brief, the JDI score was positively correlated with protein, fiber, vitamins A, C, and E, calcium, iron, potassium, and magnesium, and negatively correlated with saturated fat and sugar. Nevertheless, our previous study also showed that the JDI score was positively correlated with sodium. Furthermore, similar to the JDI, a previous study using a nationally representative dataset suggested an associations between rice and plant food and fish in the Japanese population [12], although rice (basically white rice) intake has been suggested to be related to a higher risk of diabetes both in Japan and in western countries [13, 14]. To date, except for the traditional Japanese diet, almost no dietary patterns include unhealthy dietary factors (e.g. sodium and refined grains). To investigate the nutritional characteristics of the traditional Japanese diet (which includes unhealthy dietary factors but has beneficial effects on health) would be intriguing and informative.

However, our simulation was based on the non-quantitative food frequency questionnaire method. Therefore, as a limitation, the previous study would have included considerable misclassification of foods and nutrient intakes. Additionally, as another limitation, the places of residence for the population formerly used to generate the JDI and that used in the simulation study are very close geographically, making the external validity of the simulation results still a concern. In other words, the nutritional characteristics of the traditional Japanese diet have not been sufficiently evaluated. A study using a more accurate dietary survey (such as the DR method) has therefore been needed for this purpose.

Additionally, as well as the above 9 components of the JDI, a recent systematic review has reported that several other foods, in particular, “soybeans and soybean foods”, “fruit”, and “mushrooms”, could also be applied as components of the traditional Japanese diet [15]. Therefore, there is also a need to examine the nutritional characteristics of a modified JDI that includes these 3 components.

## Methods

### Aim

The purpose of the present study was to examine the relationship between adhering to a traditional Japanese diet and nutritional characteristics. Accordingly, based on DR data, we checked the correlations between Japanese diet indices and nutrient density.

### Study participants

This was a cross-sectional study based on data from the fifth wave (July 2006 to July 2008) of the National Institute for Longevity Sciences-Longitudinal Study of Aging (NILS-LSA). Details of the NILS-LSA have been reported elsewhere [16]. In brief, participants in the NILS-LSA included randomly selected age- and sex-stratified individuals from a pool of non-institutionalized residents in the NILS neighborhood areas (Obu City, Higashiura Town) in Aichi Prefecture, Japan. The first wave of the NILS-LSA was carried out from November 1997 to April 2000 and involved 2267 participants (1139 men, 1128 women; age range 40–79 years). The participants were followed up every 2 years. When participants could not be followed up (e.g. if they moved out to the other area, dropped out for personal reasons or died), new age- and sex-matched random samples of the same number of dropout participants were recruited except for participants over 79 years old. The men and women participants aged 40 years were also newly recruited every year. In each wave, a 3-day DR was conducted for dietary assessment. We applied the data from the fifth wave in the present study for two reasons: (1) during this period, the Standard Tables of Foods Composition 2010 was applied for calculation of nutrient and energy intakes [17], and (2) the number of participants who completed the 3-day DR was the largest.

Among subjects who participated in the fifth wave (*n* = 2419, aged 40 years or over), those for whom data for the DR were unavailable were excluded (*n* = 198). Thus, a total of 2221 participants aged between 40 and 88 years were included in the present analysis.

### Dietary assessments

Dietary intakes were assessed using 3-day DRs. The DR was completed over 3 continuous days (both weekdays and 1 weekend day), and most participants completed it at home and returned records within 1 month. Food was weighed separately on a scale (1-kg kitchen scales; Sekisui Jushi, Tokyo, Japan) before being cooked or portion sizes estimated. Participants also recorded their diet using a disposable camera (27 shots; Fuji Film, Tokyo, Japan) by taking photos of meals before and after eating. Dietitians used these photos to complete missing data, and telephoned participants to resolve any discrepancies or obtain further information when necessary. Averages for 3-day nutrient and energy intakes were calculated according to the Standard Tables of Foods Composition 2010 in Japan and other sources [17].

### Japanese diet indices

In the present study, 3 Japanese diet indices were evaluated. All components of the indices were grouped based on the definitions used in the National Health and Nutrition Survey 2011 (Japan) [18].

First, according to previous studies [4, 5], we identified the JDI by the following 9 components: “rice”, “miso”, “fish and shellfish”, “green and yellow vegetables”, “seaweed”, “pickles”, “green tea”, “beef and pork”, and “coffee”. Although previous studies used the item “miso soup” (not “miso”), there was no corresponding definition in the National Health and Nutrition Survey 2011 (Japan). Therefore, “miso” (a broader definition than miso soup) was applied instead of “miso soup”. The item “beef and pork” did not include internal organs. For each of the 7 adhering components (“rice”, “miso”, “fish and shellfish”, “green and yellow vegetables”, “seaweed”, “pickles”, and “green tea”), participants were assigned 1 point if their daily intake was equal to or over the sex-specific median. For each of the 2 non-adhering components (“beef and pork” and “coffee”), participants were assigned 1 point if their daily intake was below the sex-specific median (sex-specific median was calculated using the DR data of all participants). Thus, the JDI score ranged from 0 to 9. Higher scores indicated greater conformity to the traditional Japanese diet.

Second, referring to a recent review [15], we defined a 12-component modified Japanese Diet Index (mJDI_12_) to which 3 adhering components (“soybeans and soybean foods”, “fruit”, and “mushrooms”) had been added to the JDI. Thus, the mJDI_12_ score ranged from 0 to 12.

Finally, we developed an n-component-weighted Japanese Diet Index (wJDI_n_) that comprised *n* selected components which were significantly associated with the nutrient density score from the mJDI_12_. The component selection was conducted using a multiple linear regression model and each selected component was weighted based on the results of this model.

### Nutrient density

In the present study, “nutrient density” was redefined as the percentage of daily actual intake relative to the Dietary Reference Intake (DRI) for Japanese 2015 [19]. By reference to the Nutrient-rich Foods 9.3 index [20, 21], the densities of 11 nutrients (9 components to encourage: protein, fiber, Vitamin A, Vitamin C, Vitamin E, calcium, iron, potassium, and magnesium; 2 components to limit: saturated fat and sodium) were assessed in the present study. However, our data did not include sugar because DRI for sugar is not listed in the DRIs for Japanese 2015.

The density of each nutrient was calculated using the following equation: $\mathrm{nutrient}\ \mathrm{density}=\frac{\mathrm{daily}\ \mathrm{intake}\ \mathrm{value}}{\mathrm{age}-\mathrm{sex}-\mathrm{specified}\ \mathrm{daily}\ \mathrm{reference}\ \mathrm{value}}\times 100\%$. Then, each nutrient density was further standardized by the age- and sex-specified recommended daily energy intake value (except for saturated fat). Both the daily reference value for each nutrient and the daily recommended energy intake value were based on the DRIs for Japanese, 2015 (Table 1).

**Table 1:** Daily reference values for individual nutrients and recommended energy intake values ^a^

	Unit	Sex	Daily reference value ^b^		
			30–49 years	50–69 years	≥70 years
Energy	kcal	Female	2000	1900	1750
		Male	2650	2450	2200
Protein	g	Female	50	50	50
		Male	60	60	60
Fiber	g	Female	18	18	17
		Male	20	20	19
Vitamin A ^c^	μg	Female	700	700	650
		Male	900	850	800
Vitamin C	mg	Female	100	100	100
		Male	100	100	100
Vitamin E ^d^	mg	Female	6.0	6.0	6.0
		Male	6.5	6.5	6.5
Calcium	mg	Female	650	650	650
		Male	650	700	700
Iron	mg	Female	6.5	6.5	6.0
		Male	7.5	7.5	7.0
Potassium	mg	Female	≥2600	≥2600	≥2600
		Male	≥3000	≥3000	≥3000
Magnesium	mg	Female	290	290	270
		Male	370	350	320
Sodium ^e^	mg	Female	≤2800	≤2800	≤2800
		Male	≤3200	≤3200	≤3200
Saturated fat ^f^	g	Female	< 15.6	< 14.8	< 13.6
		Male	< 20.6	< 19.1	< 17.1

^a^Guided by the Dietary Reference Intakes for Japanese, 2015

^b^Based on the Recommended Dietary Allowance (RDA). For nutrients with unavailable RDA data, the Estimated Average Requirement (EAR) or the Tentative Dietary Goal (DG) was applied

^c^Retinol equivalent

^d^Tocopherol equivalent

^e^Daily reference values for salt intake are “≤7 g for female” and “≤8 g for male”. Thus, we converted salt to sodium: Sodium (mg) = daily reference value for salt intake (g) × [600 (mg) / 1.5 (g)]

^f^Daily reference value for saturated fat is “< 7% of energy intake”. Thus, we converted saturated fat intake to volume based on “energy of saturated fatty acid =9 kcal/g”: Saturated fat (g) = daily reference value for energy intake (kcal) × 7% / 9 (kcal/g)

Thus, a nutrient density score was obtained as follows: nutrient density score =  ∑ 9 encouraged nutrient densities −  ∑ 2 limit nutrient densities; (nutrient densities were all energy-standardized except for saturated fat). A higher nutrient density score implies better overall nutrient intake.

### Statistical analysis

First, Spearman’s correlation analyses were performed to assess the correlations between the JDI score, the mJDI_12_ score and the nutrient density score.

Second, three steps were used to define the wJDI_n_: (1) because the values of the nutrient density score were not normally distributed (skewness = 1.35; kurtosis = 3.82), a logarithmic transformation of it was used (skewness = 0.25; kurtosis = 0.44). Then multiple linear regression model adjusted for energy intake was employed to select *n* significant associated components of the log nutrient density score, from the 12 components in the mJDI_12_; (2) each selected component was weighted by 10 times its standardized coefficients to obtain an integer point (for the convenience of summing up the JDI score) (standardized coefficient refers to per standard deviation increase in the predictor variable and implies the relative importance of the associations of food components with the log nutrient density score; i.e. the weight); and (3) the wJDI_n_ score was summed by the weights of all *n* selected components.

Third, Spearman’s correlation analysis was conducted to assess the correlation between the wJDI_n_ score and the nutrient density score.

To test the significance of the difference between above Spearman’s correlation coefficients (i.e. Spearman’s correlation coefficients between the JDI score, the mJDI_12_ score, the wJDI_n_ score and the nutrient density score), we calculated values of *z* for JDI, mJDI_12_, and wJDI_n_, respectively, were calculated as follows: $\mathrm{z}=1.1513\times \mathit{\lg}\frac{1+\rho }{1-\rho }$; where ρ means the Spearman’s correlation coefficients between the JDI score, the mJDI_12_ score, the wJDI_n_ score and the nutrient density score. Then ${\mathrm{S}}_{\left({\mathrm{z}}_1-{\mathrm{z}}_2\right)}=\sqrt{\frac{1}{n_1-3}+\frac{1}{n_2-3}}$ was calculated; in a same survey wave, n_1_ = n_2_ = n, so ${\mathrm{S}}_{\left({\mathrm{z}}_1-{\mathrm{z}}_2\right)}=\sqrt{\frac{2}{n_1-3}}$. Finally, ${\mathrm{U}}_{\left({\mathrm{z}}_1,{\mathrm{z}}_2\right)}=\frac{z_1-{z}_2}{{\mathrm{S}}_{\left({\mathrm{z}}_1-{\mathrm{z}}_2\right)}}$ was calculated; if ${\mathrm{U}}_{\left({\mathrm{z}}_1,{\mathrm{z}}_2\right)}$ > 1.96 or < − 1.96, then *P* < 0.05; otherwise *P* ≥ 0.05. To consider an external validity, we also assessed the correlation between the JDIs score, and between the wJDI_n_ and the nutrient density score using other period data (the sixth wave survey of the NILS-LSA; July 2008 to July 2010) (*n* = 2115).

Finally, the Spearman’s correlation was applied to assess the correlations between the 3 Japanese Diet indices scores and the 11 nutrient intakes, respectively. Energy-adjusted correlations were also checked by the residual method [22].

All data were analyzed using Statistical Analysis System (SAS) version 9.3 (SAS Institute, Cary, NC, USA). All statistical tests described here were two-sided, and differences at *P* < 0.05 were accepted as significant.

## Results

### Characteristics of participants

Our participants comprised 1104 men (49.7%) and 1117 women (50.3%), with a mean (SD) age of 60.7 (12.4) years.

### JDI, mJDI_12_ and nutrient density score

**Fig. 1:**
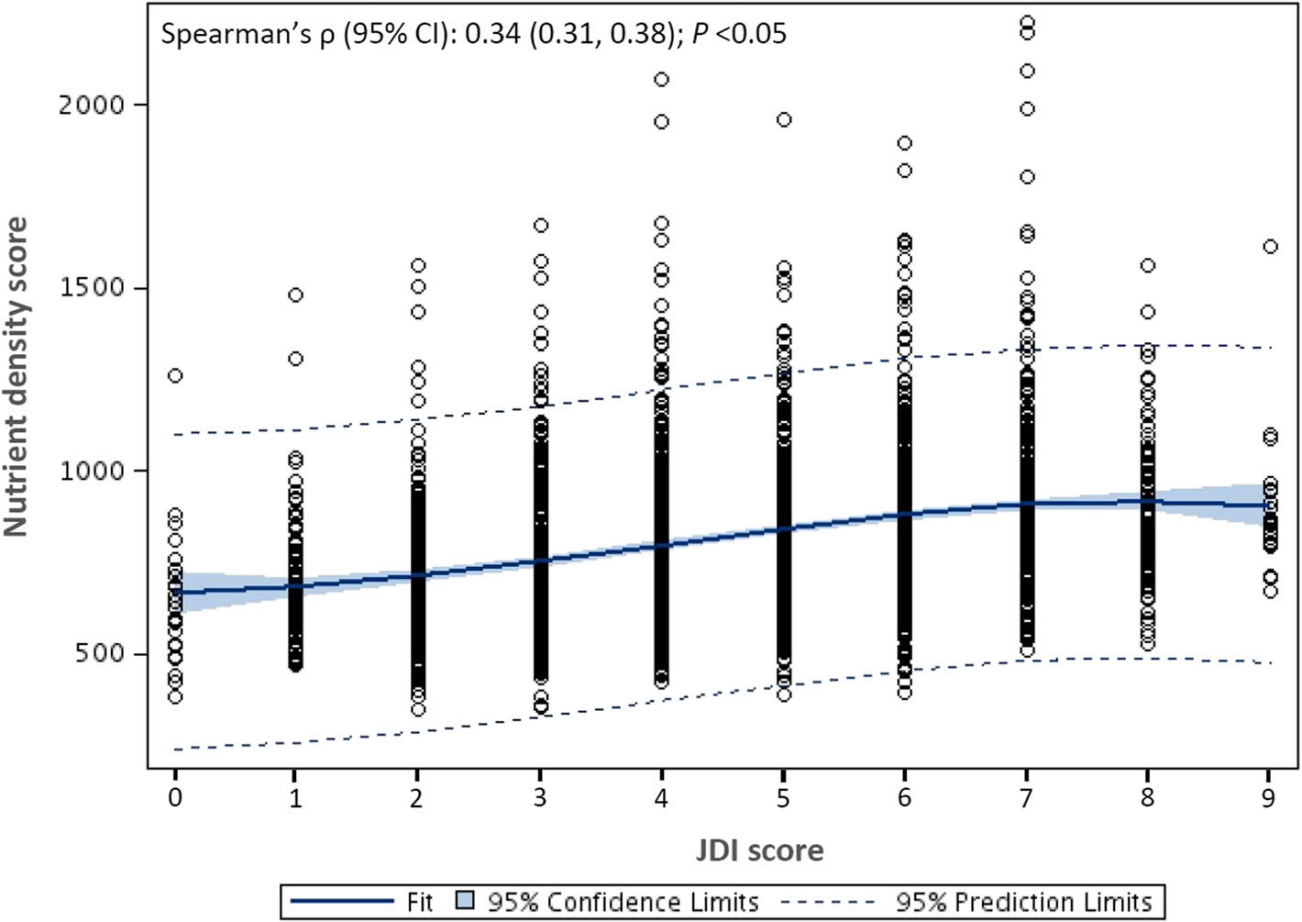
Correlation between the original 9-component Japanese Diet Index score (JDI) and the nutrient density score

**Fig. 2:**
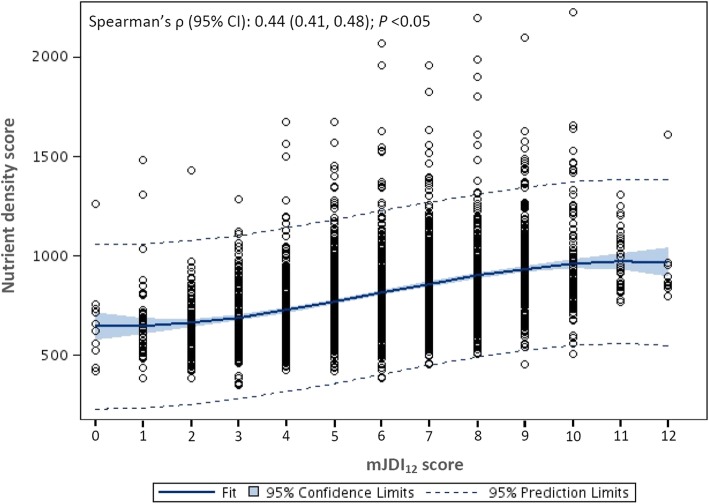
Correlation between the 12-component modified Japanese Diet Index score (mJDI_12_) and the nutrient density score

The correlations between the JDI score, the mJDI_12_ score and the nutrient density score are shown in Fig. 1 and Fig. 2, respectively. Both the JDI score and the mJDI_12_ score were positively correlated with the nutrient density score (Spearman’s ρ [95% CI], 0.34 [0.31, 0.38] for JDI score and 0.44 [0.41, 0.48] for mJDI_12_ score; both *P* < 0.05), the correlation was stronger for the mJDI_12_ score (*z* = 3.94, *P* < 0.05).

### Individual components of the mJDI_12_ and nutrient density score

The associations between individual components of the mJDI_12_ and nutrient density score are shown in Table 2. Nine components, except for miso, pickles, and coffee, were significantly correlated with the nutrient density score. Therefore, the wJDI_n_ was defined as the wJDI_9_ (9-component weighted Japanese Diet Index). Since the standardized coefficients for “green and yellow vegetables” (0.3), “fruit” (0.2) and “soybeans and soybean foods” (0.2) were higher, and the standardized coefficient for “rice” (− 0.1) was lower, the wJDI_9_ score ranged from − 1 to 12 (Additional file 1 Table S1).

**Table 2:** Associations between the components of the 12-component modified Japanese Diet Index (mJDI_12_) and the log nutrient density score (*n* = 2221)

	β ^b^	SE ^c^	*P*-value	Standardized β ^d^
Food components of the mJDI_12_ (Dummy variable) ^a^				
Rice	−0.07	0.010	< 0.001	−0.1
Miso	0.01	0.010	0.444	–
Fish and shellfish	0.04	0.009	< 0.001	0.1
Green and yellow vegetables	0.18	0.009	< 0.001	0.3
Seaweeds	0.05	0.009	< 0.001	0.1
Pickles	0.00	0.009	0.863	–
Green tea	0.07	0.009	< 0.001	0.1
Beef and pork	0.05	0.009	< 0.001	0.1
Coffee	0.01	0.009	0.437	–
Soybeans and soybean foods	0.08	0.009	< 0.001	0.2
Fruit	0.11	0.009	< 0.001	0.2
Mushrooms	0.04	0.009	< 0.001	0.1
Energy (kcal)	−0.00005	0.00001	< 0.001	

^a^Dummy variable: 0 points (reference) vs. 1 point. 0 points: daily intake <sex-specific median for rice, miso, fish and shellfish, green and yellow vegetables, seaweed, pickles, green tea, soybeans and soybean foods, fruit, and mushrooms, and daily intake ≥sex-specific median for beef and pork, and coffee; 1 point: otherwise

^b^Non-standardized regression coefficient (i.e. the expected change in the log nutrient density score when the JDI score for one food component increases from 0 to 1 while the others remain fixed)

^c^Standard error of the non-standardized regression coefficient β (i.e. an indication of how much the point estimated β is likely to vary from the corresponding population parameter)

^d^Standardized regression coefficient (referring to per standard deviation increase in the predictor variable and implying the relative importance of the relationship of food components with the log nutrient density score; i.e. the weight). Only standardized β values for food components with *P* < 0.05 are listed

### wJDI_9_ and nutrient density score

**Fig. 3:**
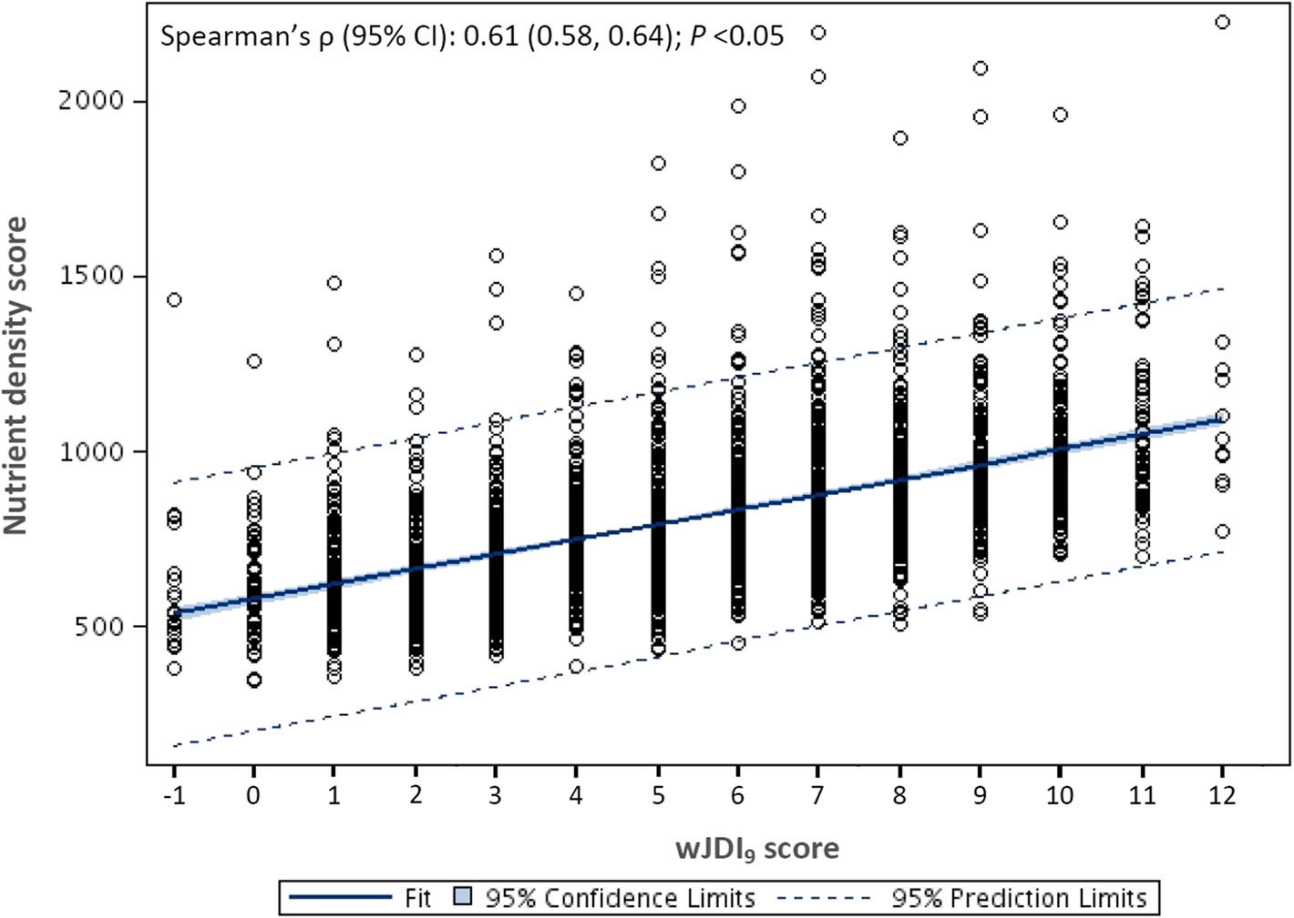
Correlation between the 9-component weighted Japanese Diet Index score (wJDI_9_) and the nutrient density score

The correlation between the wJDI_9_ score and the nutrient density score is shown in Fig. 3. The wJDI_9_ score was positively correlated with the nutrient density score (Spearman’s ρ [95% CI] = 0.61 [0.58, 0.64]; *P* < 0.05). This correlation coefficient significantly differed from those of the JDI (Spearman’s ρ [95% CI] = 0.34 [0.31, 0.38]) or the mJDI_12_ (Spearman’s ρ [95% CI] = 0.44 [0.41, 0.48]) (*z* = 11.8 and 7.9, respectively; *P* < 0.05 for both).

Even when same analyses were applied with another data (the sixth wave survey of the NILS-LSA; *n* = 2115), almost same result correlation between the wJDI_9_ score and the nutrient density score was observed (Additional file 2 Fig. S1; Spearman’s ρ [95% CI] = 0.61 [0.58, 0.64]; *P* < 0.05). This correlation coefficient was also significantly different from that of the JDI (Spearman’s ρ [95% CI] = 0.35 [0.31, 0.39]) or the mJDI_12_ (Spearman’s ρ [95% CI] = 0.44 [0.41, 0.48]) (z = 11.2 and 7.7, respectively; *P* < 0.05 for both).

### JDI, mJDI_12_, wJDI_9_ score and nutrient intakes

The correlations between the JDI, mJDI_12_, wJDI_9_ score and nutrient intakes (components of the nutrient density score) are shown in Table 3. In the energy-adjusted results, all of the scores for these 3 indices were positively correlated with the intakes of all 9 encouraged nutrients (range of Spearman’s ρ, 0.15–0.49 for the JDI, 0.21–0.55 for the mJDI_12_, 0.36–0.66 for the wJDI_9_), and negatively correlated with saturated fat intake (corresponding Spearman’s ρ, − 0.35, − 0.32, and − 0.16, respectively). However, the scores for all of these 3 indices were positively correlated with sodium intake (corresponding Spearman’s ρ, 0.38, 0.38, and 0.26, respectively). All of the above correlations were statistically significant (*P* < 0.05).

**Table 3:** Correlations between JDI, mJDI_12_, wJDI_9_ score ^a^ and nutrient intakes (n = 2221)

	Crude						Energy-adjusted					
	JDI		mJDI_12_		wJDI_9_		JDI		mJDI_12_		wJDI_9_	
Energy (kcal)	0.09	(0.05, 0.13)	0.12	(0.08, 0.16)	0.06	(0.02, 0.10)						
Protein (g)	0.23	(0.19, 0.27)	0.28	(0.24, 0.32)	0.26	(0.22, 0.30)	0.26	(0.22, 0.30)	0.31	(0.27, 0.35)	0.36	(0.32, 0.40)
Fiber (g)	0.42	(0.39, 0.46)	0.55	(0.51, 0.58)	0.63	(0.60, 0.65)	0.42	(0.38, 0.45)	0.54	(0.50, 0.57)	0.65	(0.62, 0.67)
Vitamin A (μg)	0.28	(0.24, 0.32)	0.35	(0.31, 0.39)	0.45	(0.41, 0.48)	0.25	(0.21, 0.29)	0.31	(0.27, 0.35)	0.43	(0.39, 0.47)
Vitamin C (mg)	0.34	(0.30, 0.38)	0.42	(0.39, 0.46)	0.49	(0.45, 0.52)	0.31	(0.27, 0.34)	0.38	(0.34, 0.42)	0.46	(0.43, 0.50)
Vitamin E (mg)	0.18	(0.14, 0.22)	0.25	(0.21, 0.29)	0.38	(0.34, 0.42)	0.15	(0.11, 0.19)	0.21	(0.17, 0.25)	0.41	(0.37, 0.45)
Calcium (mg)	0.26	(0.22, 0.30)	0.34	(0.30, 0.38)	0.43	(0.40, 0.47)	0.23	(0.19, 0.27)	0.31	(0.27, 0.35)	0.44	(0.40, 0.47)
Iron (mg)	0.46	(0.43, 0.50)	0.53	(0.50, 0.56)	0.50	(0.47, 0.53)	0.49	(0.45, 0.52)	0.55	(0.52, 0.59)	0.56	(0.52, 0.59)
Potassium (mg)	0.37	(0.33, 0.41)	0.49	(0.45, 0.52)	0.60	(0.57, 0.62)	0.38	(0.34, 0.41)	0.49	(0.46, 0.53)	0.66	(0.64, 0.69)
Magnesium (mg)	0.39	(0.35, 0.43)	0.49	(0.46, 0.52)	0.54	(0.51, 0.57)	0.42	(0.39, 0.46)	0.53	(0.50, 0.56)	0.64	(0.62, 0.67)
Sodium (mg)	0.36	(0.33, 0.40)	0.37	(0.34, 0.41)	0.25	(0.21, 0.29)	0.38	(0.35, 0.42)	0.38	(0.34, 0.41)	0.26	(0.22, 0.30)
Saturated fat (g)	−0.23	(−0.27, −0.19)	−0.19	(−0.23, −0.15)	−0.09	(−0.14, −0.05)	−0.35	(−0.39, −0.31)	−0.32	(−0.36, −0.28)	−0.16	(−0.20, −0.12)
Saturated fatty acid, % ^c^	−0.17	(− 0.21, − 0.12)	−0.15	(− 0.19, − 0.11)	−0.07	(− 0.11, − 0.03)						
Sodium / Potassium	−0.03	(−0.07, 0.01)	− 0.15	(− 0.19, − 0.10)	−0.38	(− 0.42, − 0.34)						

^a^JDI, Japanese Diet Index (original 9-component Japanese Diet Index); mJDI_12_, 12-component modified Japanese Diet Index; wJDI_9_, 9-component weighted Japanese Diet Index; CI, confidence interval

^b^Components of the nutrient density score: 9 encouraged nutrients (protein, fiber, vitamin A, vitamin C, vitamin E, calcium, iron, potassium, magnesium) and 2 limited nutrients (sodium, saturated fat)

^c^Saturated fatty acid (g)/all fatty acid (g) × 100%

Median values for nutrient intakes according to the tertiles of the JDI, mJDI_12_, and wJDI_9_ are shown in Additional file 1 Table S2, the associations between scores and nutrient intakes were similar to those in Table 3.

## Discussion

The purpose of the present study was to examine the relationship between adhering to a traditional Japanese diet and nutrient density. Based on data from a DR survey of a large sample (*n* = 2221), correlations between the scores for 3 Japanese diet indices (JDI, mJDI_12_, wJDI_9_) and nutrient density were investigated. A higher JDI score was shown to be correlated with a higher nutrient density score.

The positive correlation between the mJDI_12_ (3 food components, i.e. “soybeans and soybean foods”, “fruit”, and “mushrooms”, added to the JDI) score and the nutrient density score was stronger than that for the JDI. Additionally, the positive correlation between the wJDI_9_ score (summed by weighted points for 9 selected components, excluding “miso”, “pickles”, and “coffee” from the mJDI_12_) and the nutrient density score was stronger than that for the mJDI_12_ (corresponding Spearman’s ρ, 0.61 vs. 0.44). These results suggest that the mJDI_12_ or the wJDI_9_ is more useful for defining better overall nutrient intakes than the JDI. For each nutrient component of the nutrient density score, intakes of magnesium, potassium, fiber and calcium were better according to the mJDI_12_ than according to the JDI. Because soybeans and soybean foods [23], fruit [24], and mushrooms [25] are known to be resources of these nutrients, these food components would have contributed to improving the nutrient intakes.

However, the scores for all 3 indices were positively correlated with the intake of sodium, being consistent with a previous study [8]. After excluding “miso” and “pickles” (which are known to have a high salt content) from the mJDI_12_, although the correlation between the wJDI_9_ score and sodium intake became weaker, a considerable correlation remained. A previous study has reported that seasonings such as soy sauce and salt are the major sources contributing to total sodium intake in Japan [26]. In the present study, seasonings were also ranked in the top 3 (Additional file 1 Table S4). On the other hand, scores for all 3 indices were positively correlated with potassium intake, and inversely correlated with the sodium/potassium ratio (especially for the wJDI_9_), also being consistent with the previous study [8]. Thus, for the traditional Japanese diet, a high potassium intake might offset the demerit of high sodium intake.

Based on the association between each component of the mJDI_12_ and the nutrient density score, we developed a weighted index, the “wJDI_9_”, which encourages a higher intake of “green and yellow vegetables”, “soybeans and soybean foods” and “fruit”, and a lower intake of “rice” (even the traditional Japanese diet is commonly thought to be rice-centered). The previous study had suggested that a dietary pattern with similar weights contributed to higher overall nutrient adequacy and lower oxidative stress [8]. Another study has also indicated that a Japanese food score that did not encourage rice intake was related to lower cardiovascular disease mortality [1]. These findings suggest that, although rice is a staple food in Japan, a diet that includes excessive and single intake of rice may not be desirable. As a post hoc analysis, we checked food intake volumes among better cases (participants who were in both the top 25% for nutrient density score and top 25% for the wJDI_9_ score) (Additional file 1 Table S3). As a result, the mean daily intake volume of “rice” was 278 g (mean daily total energy intake, 2013 kcal), being rather lower than that for the Japanese population as a whole (corresponding values, 317 g for the 50–59-year age group [1916 kcal], 310 g for the 60–69-year age group [1892 kcal], and 323 g for the ≥70-year age group [1745 kcal]) [18].

It has often been assumed that the traditional Japanese diet is a lean diet such as that of Buddhist cuisine (*shojin-ryori* in Japanese), which might contribute to the prevention of obesity [27]. On the other hand, a lean diet (which is usually inadequately defined in terms of nutrient and food content, and contains less red meat and fat) might also contribute to undernutrition [28]. However, since all indices were related to sufficient intakes of energy and protein (Table 3 and Additional file 1 Table S2), the traditional Japanese diet as defined in the present study may not contribute to a high risk of undernutrition.

Indices have been proven to be useful in epidemiological studies and to develop and apply nutritional strategies [29] and some dietary indices (such as the healthy eating index and Mediterranean diet index) have been widely applied in western populations. Given the specific situation of diet intake in Japan (a comparable lower total calorie intake, a different Protein-Fat-Carbohydrate balance, and a different fatty acid ratio compared with western countries [30–33]), development, modification, and application of the JDI may be important for public health promotion for the Japanese population. However, it is still worth noting that, among the practical aspects of our finding, comprehensive health consultation and guidance should be conducted involving other lifestyle behaviors (e.g. physical activity), socioeconomic context (e.g. sociocultural habits, religious beliefs), food access (e.g. food handling, preparation and storage, purchase of seasonal and local foods,) and so on [34].

This study had several limitations. First, as a general limitation of the DR method, a certain number of subjects might have reported foods that are considered healthier and more socially desirable than what they usually eat [35]. However, since we focused on the dietary pattern (e.g. combinations of food intake), this potential pitfall may not have had a significant impact on our findings. Second, because the DR method is dependent on food composition databases, nutrient intake might have been misclassified in the present study. For example, participants who were conscious of their salt intake might have been more likely to select low-salt versions of some foods that are generally considered to be high in salt (e.g. miso, pickles). If the present study included a substantial fraction of such individuals, then the absolute sodium intake volume and the subsequent correlation between the Japanese Diet Indices score and sodium intake might have been overestimated. Third, although participants used for validation (2008–2010) were not totally the same as those used for the main analyses (2006–2008), they were collected from the same region. Additionally, the National Health and Nutrition Surveys in 2006 [36] and 2017 [37] indicated that the average consumption of “fish and shellfish”, and “green and yellow vegetables” decreased, and the average consumption of “beef and pork” increased during the recent 10 years (the Westernization of the Japanese diet [38]). Thus, the average higher consumption of these food components by our participants would represent a relatively traditional Japanese diet in Japan. A further validation study would be needed with participants in a different context. Finally, the associations between JDIs and biomarkers were not assessed in the present study. It would be informative to investigate this issue in the future to confirm the nutritional characteristics of the traditional Japanese diet.

## Conclusions

Adhering to a traditional Japanese diet as defined by the JDI was associated with good nutrient density. Furthermore, the modified indices (mJDI_12_ and wJDI_9_) had higher performance in terms of nutrient density. However, as a demerit, these indices were correlated with high sodium intake.

## Additional files

Additional file 1: **Table S1.** Points for calculation of the 9-component weighted Japanese Diet index (wJDI_9_). **Table S2.** Nutrient intakes according to JDI, mJDI_12_, wJDI_9_ score (*n* = 2221). **Table S3.** Food intakes among the better cases (*n* = 315). **Table S4.** Percentage of sodium intake from each food in the total diet (n = 2221). (DOCX 47 kb)

Additional file 2: **Figure S1.** Correlation between the 9-component weighted Japanese Diet Index score (wJDI_9_) and the nutrient density score (applied with the sixth wave survey of the NILS-LSA; *n* = 2115. (PDF 263 kb)

## Data Availability

The data that support the findings of this study are available from the National Institute for Longevity Sciences-Longitudinal Study of Aging (NILS-LSA) but restrictions apply to the availability of these data, which were used under license for the current study, and so are not publicly available. Data are however available from the authors upon reasonable request and with permission of the NILS-LSA.

## Abbreviations

JDI: Japanese diet index
mJDI_12_: 12-component modified index
NILS-LSA: National Institute for longevity sciences-longitudinal study of aging
wJDI_9_: weighted Japanese diet index
