# Supplementary material for: A cross-sectional study of the associations between the traditional Japanese diet and nutrient intakes: the NILS-LSA project
Source: Nutr J. 2019 Jul 30;18:43. doi: 10.1186/s12937-019-0468-9 (PMC6664518; doi:10.1186/s12937-019-0468-9)
Supplement: Supplementary file 1 — Table S1. Points for calculation of the 9-component weighted Japanese Diet index (wJDI9). Table S2. Nutrient intakes according to JDI, mJDI12, wJDI9 score (n = 2221). Table S3. Food intakes among the better cases (n = 315). Table S4. Percentage of sodium intake from each food in the total diet (n = 2221). (DOCX 47 kb) [file 12937_2019_468_MOESM1_ESM.docx]

| **Additional file 1** **Table 1**. Points for calculation of the 9-component weighted Japanese Diet index (wJDI_9_). | | | |
| --- | --- | --- | --- |
| Food components of wJDI_9_ | | Point (weight) | |
|  |  | Daily intake < sex-specific median | Daily intake ≥ sex-specific median |
|  | Rice | 0 | -1 |
|  | Green and yellow vegetables | 0 | 3 |
|  | Fruit | 0 | 2 |
|  | Soybeans and soybean foods | 0 | 2 |
|  | Fish and shellfish | 0 | 1 |
|  | Seaweeds | 0 | 1 |
|  | Green tea | 0 | 1 |
|  | Mushrooms | 0 | 1 |
|  | Beef and pork | 1 | 0 |
|  | |  | |
| Range of the wJDI_9_ score | | -1 to 12 | |

| **Additional file 1** **Table 2**. Nutrient intakes according to JDI, mJDI_12_, wJDI_9_ ^a^ score (n =2221). | | | | | | | | | | |
| --- | --- | --- | --- | --- | --- | --- | --- | --- | --- | --- |
|  | | Median values of nutrient intakes (per day) | | | | | | | | |
|  |  | Tertiles of the  JDI score | | | Tertiles of the  mJDI_12_ score | | | Tertiles of the  wJDI_9_ score | | |
|  |  | Lower | Middle | Higher | Lower | Middle | Higher | Lower | Middle | Higher |
| Energy (kcal) | | 1951 | 1985 | 2019 | 1943 | 1990 | 2029 | 1964 | 1998 | 1990 |
|  | |  |  |  |  |  |  |  |  |  |
| Protein (g) | | 71.0 | 76.0 | 78.5 | 69.7 | 75.7 | 79.6 | 70.1 | 76.2 | 78.5 |
| Fiber (g) | | 13.2 | 15.5 | 17.7 | 12.7 | 15.5 | 18.6 | 12.5 | 15.5 | 18.9 |
| Vitamin A (μg) ^b^ | | 412.6 | 511.4 | 586.7 | 384.5 | 504.7 | 614.6 | 370.7 | 497.2 | 637.2 |
| Vitamin C (mg) | | 87.7 | 110.7 | 136.0 | 83.6 | 110.9 | 145.1 | 78.3 | 111.6 | 148.7 |
| Vitamin E (mg) ^c^ | | 8.2 | 8.7 | 9.4 | 7.9 | 8.7 | 9.6 | 7.6 | 8.7 | 9.9 |
| Calcium (mg) | | 504.1 | 586.3 | 622.2 | 479.6 | 587.5 | 642.8 | 477.2 | 574.8 | 661.3 |
| Iron (mg) | | 7.6 | 8.8 | 10.2 | 7.4 | 9.2 | 10.4 | 7.6 | 8.9 | 10.1 |
| Potassium (mg) | | 2349 | 2669 | 2896 | 2244 | 2676 | 3026 | 2180 | 2682 | 3095 |
| Magnesium (mg) | | 248.9 | 280.9 | 314.7 | 239.4 | 283.3 | 323.2 | 238.3 | 284.2 | 320.4 |
| Sodium (mg) | | 3897 | 4251 | 4687 | 3845 | 4251 | 4734 | 4022 | 4243 | 4533 |
| Saturated fat (g) | | 16.4 | 14.8 | 13.6 | 16.4 | 14.8 | 13.7 | 15.9 | 14.8 | 14.6 |
| ^a^ | JDI, Japanese Diet Index (original 9-component Japanese Diet Index); mJDI_12_, 12-component modified Japanese Diet Index; wJDI_9_, 9-component weighted Japanese Diet Index. | | | | | | | | | |
| ^b^ | Retinol equivalent. | | | | | | | | | |
| ^c^ | Tocopherol equivalent. | | | | | | | | | |

| **Additional file 1** **Table 3**. Food intakes among the better cases (n =315) ^a^. | | | |
| --- | --- | --- | --- |
|  | | Mean | SD ^b^ |
| Food intakes (g/day) | |  | |
|  | Rice | 278 | 121 |
|  | Miso | 14 | 8 |
|  | Fish and shellfish | 106 | 51 |
|  | Green and yellow vegetables | 178 | 78 |
|  | Seaweeds | 31 | 35 |
|  | Pickles | 22 | 23 |
|  | Green tea | 547 | 419 |
|  | Beef and pork | 37 | 30 |
|  | Coffee | 95 | 134 |
|  | Soybeans and soybean foods | 102 | 81 |
|  | Mushrooms | 18 | 16 |
|  | Fruit | 205 | 113 |
|  | Other vegetables | 200 | 88 |
|  | Potatoes | 53 | 41 |
|  | Eggs | 41 | 24 |
|  | Dairy products | 181 | 133 |
|  | Soy sauce | 18 | 10 |
|  |  |  |  |
| Energy (kcal/day) | | 2013 | 383 |
| ^a^ | Participants who were in both the top 25% for nutrient density score and top 25% for the wJDI_9_ score. | | |
| ^b^ | SD, standard deviation. | | |

| **Additional file 1** **Table 4**. Percentage of sodium intake from each food in the total diet (n =2221) ^a^. | | |
| --- | --- | --- |
|  | | Sodium intake (% / day) |
| Food | |  |
|  | Soy sauce | 20.4 |
|  | Other seasonings ^b^ | 17.4 |
|  | Miso | 12.1 |
|  | Table salt | 7.8 |
|  | Bread | 4.9 |
| ^a^ | Top five only. | |
| ^b^ | Such as *mentsuyu* (usually made of soy sauce, sugar, *mirin* and fish stock), soup stocks, tomato ketchup, curry roux, dressings, *mirin* (a mixture of sugar and a 14% alcohol), consommé cubes, vinegars, etc. | |
