# Supplementary figures and images for: A cross-sectional study of the associations between the traditional Japanese diet and nutrient intakes: the NILS-LSA project
Source: Nutr J. 2019 Jul 30;18:43. doi: 10.1186/s12937-019-0468-9 (PMC6664518; doi:10.1186/s12937-019-0468-9)

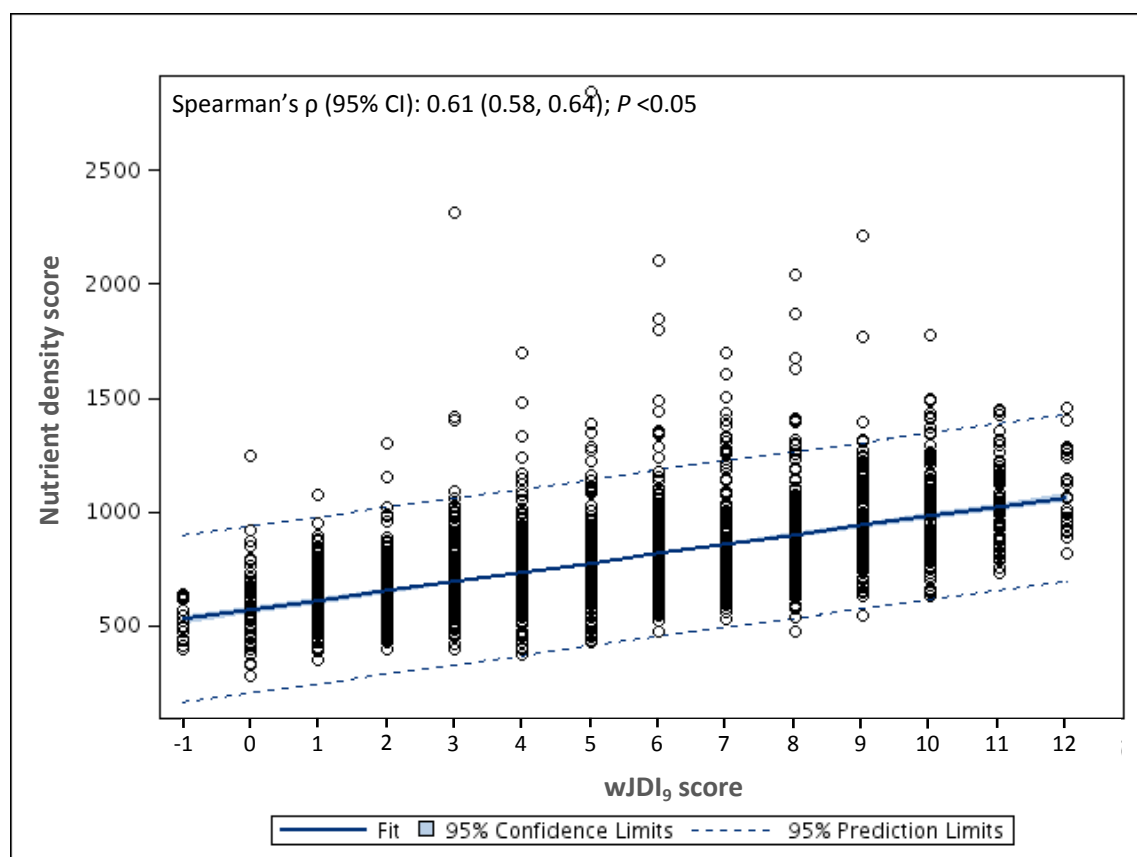

Supplement: Supplementary file 2 — Figure S1. Correlation between the 9-component weighted Japanese Diet Index score (wJDI9) and the nutrient density score (applied with the sixth wave survey of the NILS-LSA; n = 2115. (PDF 263 kb) [file 12937_2019_468_MOESM2_ESM.pdf]
